# A Novel Broad Host Range Phage Infecting *Alteromonas*

**DOI:** 10.3390/v13060987

**Published:** 2021-05-26

**Authors:** Xuejin Feng, Wei Yan, Anan Wang, Ruijie Ma, Xiaowei Chen, Ta-Hui Lin, Yi-Lung Chen, Shuzhen Wei, Tao Jin, Nianzhi Jiao, Rui Zhang

**Affiliations:** 1 State Key Laboratory of Marine Environmental Science, Fujian Key Laboratory of Marine Carbon Sequestration, College of Ocean and Earth Sciences, Xiamen University, Xiamen 361102, China; fengxj99@stu.xmu.edu.cn (X.F.); yanwei@cug.edu.cn (W.Y.); 18206078261@163.com (A.W.); maruijie@stu.xmu.edu.cn (R.M.); chenxwad@gmail.com (X.C.); l161110@xmu.edu.cn (T.-H.L.); yilungchen@scu.edu.tw (Y.-L.C.); weisz@stu.xmu.edu.cn (S.W.); 2 College of Marine Science and Technology, China University of Geosciences, Wuhan 430074, China; 3 Guangzhou Magigene Biotechnology Co., Ltd., Guangdong 510000, China; jintao@magigene.com; 4 Southern Marine Science and Engineering Guangdong Laboratory (Zhuhai), Zhuhai 519080, China

**Keywords:** *Alteromonas*, phage, *Autographiviridae*, host range

## Abstract

Bacteriophages substantially contribute to bacterial mortality in the ocean and play critical roles in global biogeochemical processes. *Alteromonas* is a ubiquitous bacterial genus in global tropical and temperate waters, which can cross-protect marine cyanobacteria and thus has important ecological benefits. However, little is known about the biological and ecological features of *Alteromonas* phages (alterophages). Here, we describe a novel alterophage vB_AmeP-R8W (R8W), which belongs to the *Autographiviridae* family and infects the deep-clade *Alteromonas mediterranea*. R8W has an equidistant and icosahedral head (65 ± 1 nm in diameter) and a short tail (12 ± 2 nm in length). The genome size of R8W is 48,825 bp, with a G + C content of 40.55%. R8W possesses three putative auxiliary metabolic genes encoding proteins involved in nucleotide metabolism and DNA binding: thymidylate synthase, nucleoside triphosphate pyrophosphohydrolase, and PhoB. R8W has a rapid lytic cycle with a burst size of 88 plaque-forming units/cell. Notably, R8W has a wide host range, such that it can infect 35 *Alteromonas* strains; it exhibits a strong specificity for strains isolated from deep waters. R8W has two specific receptor binding proteins and a compatible holin–endolysin system, which contribute to its wide host range. The isolation of R8W will contribute to the understanding of alterophage evolution, as well as the phage–host interactions and ecological importance of alterophages.

## 1. Introduction

Viruses are widely distributed in the oceans, with an average abundance of approximately 10^7^ viruses mL^−1^ at the ocean surface [1]. Most marine viruses are bacteriophages that infect bacteria and cause an estimated bacterial mortality rate of 10–50% daily in the ocean [1]. Bacteriophages are exceptionally diverse, both morphologically and genetically; they can facilitate horizontal gene transfer and hence are important for bacterial diversity and evolution [2]. In addition, phage-encoded auxiliary metabolic genes (AMGs), such as fatty acid desaturase genes to regulate the fluidity of host membranes, can enhance phage fitness [3,4,5,6]. Therefore, bacteriophages play crucial roles in marine ecosystems and global biogeochemical cycles [7,8].

The marine bacteria *Alteromonas*, a genus within the Gammaproteobacteria belonging to a Gram-negative organism, is widely distributed in global tropical and temperate waters [9]. Thus far, 28 species of *Alteromonas* have been identified. *Alteromonas* can incorporate the transient nutrients released from phytoplankton to grow rapidly in oligotrophic open oceans [10]. Importantly, although *Alteromonas* spp. are not always abundant in the environment, they demonstrate high catalase activity and thus can remove hydrogen peroxide from the ocean [11]. Furthermore, *Alteromonas* can act as important helper bacteria that cross-protect marine cyanobacteria, the most abundant phytoplankton in oligotrophic oceans [12,13]. Hence, *Alteromonas* spp. are actively involved in the circulation of biogenic matter and the flow of energy in the ocean [12,13].

Despite the ecological importance of the *Alteromonas* genus, investigations of phages that infect *Alteromonas* (alterophages) to unveil their genomic features and potential impacts on host bacteria have lagged considerably behind analyses of other marine phages [14,15,16,17,18,19]. For example, more than 100 cyanophages have been published and characterized [20]. Studies of cyanophages and cyanophage–cyanobacteria interactions have greatly advanced the overall understanding of microbial oceanographic processes [4,21]. There are currently 12 published genomes of cultivated marine alterophages, using seven *Alteromonas* species as hosts (Table 1) [14,15,16,17,18,19]. This limited information regarding alterophages has impeded the overall understanding of the biological and ecological importance of alterophages.

A well-studied model *Alteromonas* species, *Alteromonas macleodii*, can be divided into two clades based on physiological and genomic characteristics: surface (*A. macleodii*) and deep (*Alteromonas mediterranea*), which are found in ocean surface and deep waters, respectively [22]. In this study, we used the typical deep-clade *A. mediterranea* as the host to isolate and analyze the novel alterophage vB_AmeP-R8W (R8W). R8W comprises the second known member of the *Foturvirus* genus within the *Autographiviridae* family. We carried out a comprehensive survey of host range and performed analyses involving classification and genomic organization of R8W. Importantly, we found that R8W contains various genes that may broaden its host range and affect alterophage–*Alteromonas* interactions.

## 2. Materials and Methods

### 2.1. Phage Isolation, Purification, and Amplification

R8W was isolated from coastal seawater using the double-layer agar method [23]. The water samples were collected from Xiamen Bay (latitude *N* = 24.253, longitude *E* = 118.014, depth = 3 m) in October 2014 (Supplementary Materials Figure S1), then filtered through a 0.22 μm membrane (Millipore, Bedford, MA, USA) to remove bacteria and stored in the dark at 4 °C. Host strain *A. mediterranea* DE^T^ was a type strain (DSM17117^T^ = CIP 110805^T^ = LMG 28347^T^), previously isolated from a depth of 1000 m in the Mediterranean Sea [24]. The host strain was cultured at 28 °C with shaking at 180 rpm in RO medium (200 mL of filtered seawater and 800 mL of artificial seawater supplemented with 0.1% yeast extract, 0.1% peptone, 0.1% sodium acetate, and 0.1% trace metal solution (1 × 10^−5^ M FeCl_3_•6H_2_O, 1 × 10^−5^ M Na_2_EDTA•2H_2_O, 4 × 10^−8^ M CuSO_4_•5H_2_O, 3 × 10^−8^ M Na_2_MoO_4_•2H_2_O, 8 × 10^−8^ M ZnSO_4_•7H_2_O, 5 × 10^−8^ M CoCl_2_•6H_2_O, and 9 × 10^−7^ M MnCl_2_•4H_2_O)) [23]. Subsequently, 100 mL of host culture was inoculated with the seawater overnight to enrich phages [23]. Thereafter, the phage was filtered and mixed with *A. mediterranea* DE using the double-layer agar method. After overnight co-culture, clonal plaques were picked from the lawn of host cells and added to 1 mL of SM buffer (50 mM Tris-HCl, 0.1 M NaCl, 8 mM MgSO_4_, pH 7.5) [23]. These steps were repeated five times to purify the phage. Before DNA extraction, 1 L phage lysate was treated with 2 mg/L of DNase I and RNase A for 1 h at 25 °C, then supplemented with 1 M NaCl for 30 min at 4 °C to promote the separation of phage particles and cell debris. The phage suspension was harvested by centrifugation (10,000× *g* for 10 min at 4 °C) and passed through 0.22 μm filters, then mixed with 10% polyethylene glycol (PEG8000) and stored for 24 h at 4 °C. The mixture was then centrifuged (10,000× *g* for 1 h at 4 °C) to precipitate the phage particles. The phage particles were resuspended in 6 mL of SM buffer. High-titer phage suspensions were prepared via CsCl (1.3%, 1.5%, and 1.7%) density gradient centrifugation (200,000× *g* for 24 h at 4 °C), followed by 30 kDa centrifugal filtration (Millipore, Bedford, MA, USA). The filtrate was stored at 4 °C for subsequent experiments.

### 2.2. Cross-Infection Experiments

Cross-infections involving phage R8W were performed using 79 *Alteromonas* strains (see detailed information in Supplementary Materials Table S1). Host cultures (1 mL) undergoing exponential growth were mixed with 5 mL of RO agar medium (0.5% agar, cooled to 45 °C) and poured onto a solid plate of 1.5% RO agar medium. After the agar had solidified, a 1:100 dilution of the phage lysate was aliquoted onto the host lawn (5 μL aliquots) and incubated at 28 °C overnight; SM buffer alone was used as a blank control. Each plaque formation was regarded as successful infection of the corresponding tested strain by R8W.

### 2.3. One-Step Growth Curve

At a 0.01 multiplicity of infection, the purified phages were adsorbed to 1 mL of exponentially growing host cells in the dark at room temperature for 10 min. Free phages were removed by centrifugation (10,000× *g* for 5 min at 4 °C). Samples were then resuspended in 100 mL of RO medium for sample collection at 15-min intervals for a total duration of 135 min. Plaques were counted with the double-layer plate method [23]. The co-culture of R8W and host were incubated at 28 °C overnight.

### 2.4. Transmission Electron Microscopy (TEM)

Phage morphology was analyzed by TEM. Briefly, 20 μL of high-titer phage concentrate was placed on 200-mesh formvar-coated copper electron microscopy grids and allowed to adsorb for 20 min. The phage was negatively stained with 1% (*w*/*v*) phosphotungstic acid for 1 min; the excess stain was removed with filter paper and then air dried for 2 h. The phage was imaged using a Tecnai G2 Spirit BioTwin TEM (FEI Thermo Fisher Scientific, Eindhoven, The Netherlands) at 120 kV.

### 2.5. Genome Sequencing, Assembly and Annotation

The phage was lysed by incubation with 50 μM proteinase K, 20 mM EDTA, and 0.5% SDS at 65 °C for 3 h. The phage DNA was extracted using the phenol-chloroform method. DNA was sonicated using a Covaris to an average length of 350 bp. DNA fragments were then end repaired, 3′-adenylated, and amplified using Illumina sequencing adapter-specific primers. After quality control, quantification and normalization of the DNA libraries, 150 bp paired-end reads were generated from the Illumina Novaseq. Raw reads were trimmed using Trimmomatic version 0.36 [25] (parameters: version 0.36, ILLUMINACLIP: TruSeq3-PE.fa:2:30:10 LEADING:3 TRAILING:3 SLIDINGWINDOW:4:15 MINLEN:40) to gain clean reads, which comprised more than 90% of the raw data. Bowtie2 version 2.3.4 [26] was used to remove sequences attributed to the host bacterial genome; high-quality clean reads were then assembled using IDBA-UD version 1.1.3 (parameters: kmer min 21 max 91 step 10) [27]. The termini were identified by PhageTerm [23,28]. The reads with the maximum coverage were considered as phage termini, which was “5′-CTGGGCACTAACCCACACAACGTACCATAT-3′”. The complete genome sequence was submitted to the GenBank database under accession number MW043865.

The genes in assembled genomic sequences were predicted by PRODIGAL [29], GeneMarkS [30] (http://topaz.gatech.edu/GeneMark/genemarks.cgi, accessed on 9 April 2021), and RAST (http://rast.nmpdr.org/, accessed on 9 April 2021) [31]. Annotation of the functions of the predicted gene products were conducted using BLAST search algorithms (parameters: e-value <0.001). The tRNAscan-SE program was used to predict tRNA sequences [32].

The receptor binding proteins (RBPs) were identified using a previously described method [33]. The functions of the predicted R8W gene products that were annotated as tail fibers, tail spikes, and hypothetical proteins were analyzed using Phyre2 (http://www.sbg.bio.ic.ac.uk/phyre2/html/page.cgi?id=index, accessed on 9 April 2021) (parameters: confidence > 40%) [34], UniProtKB (https://www.uniprot.org, accessed on 9 April 2021) (parameters: e-value < 0.01), Pfam (http://pfam.xfam.org/search/sequence, accessed on 9 April 2021) (parameters: e-value < 0.01) [35], and HHPred (https://toolkit.tuebingen.mpg.de, accessed on 9 April 2021) (parameters: e-value < 0.001, cols > 80) to predict depolymerase activity [36]. The structures were predicted using the I-TASSER server (https://zhanglab.ccmb.med.umich.edu/I-TASSER/, accessed on 9 April 2021) and visualized using PyMOL [37,38].

### 2.6. Phylogenetic Analysis

The large terminase subunit (TerL) and thymidylate synthase (ThyX and ThyA) proteins were used for phylogenetic analysis. The TerL protein is conserved in Caudovirales [39]. ThyX and ThyA are encoded by putative AMG and are present in many viral and bacterial genomes [40]. Phylogenetic analyses of these two proteins were used to assess the genetic distances of members within the *Autographiviridae*. Individual amino acid sequences of the proteins were aligned using Mafft version 7.313 [41] (parameters: –adjustdirectionaccurately –auto), and a maximum-likelihood phylogenetic tree was constructed using RAxML version 8.2.11 with a bootstrap value of 1000 (parameters: -f a -m PROTGAMMAWAG -N 1000) [42]. The average nucleotide identity (ANI) was calculated using OrthANI software [43] and JSpeciesWS (http://jspecies.ribohost.com/jspeciesws/#analyse, accessed on 9 April 2021) [44] to establish ANI phylogenetic trees.

The classification of R8W among 57 reference genomes (i.e., most currently known *Autographiviridae* viruses, 12 alterophages (see detailed information in Table 1), and other similar viruses) was performed using the Virus Classification and Tree Building Online Resource (VICTOR, https://victor.dsmz.de, accessed on 9 April 2021) as described by Meier-Kolthoff and colleagues [45,46]. Briefly, all pairwise comparisons of viral nucleotide sequences were conducted using the genome BLAST distance phylogeny method under settings recommended for prokaryotic viruses [46,47]. The taxonomies at levels of species, genus, subfamily, and family were estimated using the OPTSIL program with the recommended clustering thresholds and an F-value (fraction of links required for cluster fusion) of 0.5 [45,46].

## 3. Results and Discussion

### 3.1. Biological Characteristics

**Figure 1:**
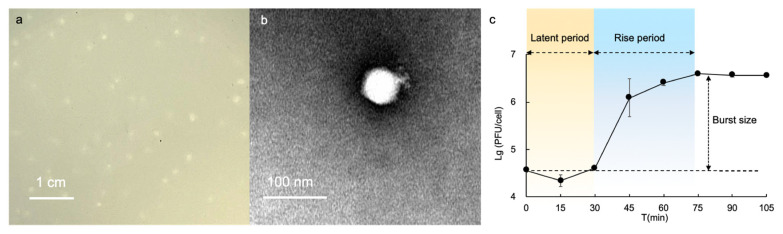
Biological characteristics of the alterophage vB_AmeP-R8W (R8W). (**a**) Image of plaques 24 h after infection. (**b**) TEM of the podovirus alterophage R8W. (**c**) One-step growth curve of R8W in the host, *Alteromonas mediterranea* strain DE. Error bars indicate standard deviation.

A novel alterophage R8W was isolated from Xiamen Bay, China, using the deep-clade *A. mediterranea* DE as a host. After 24 h of infection with purified R8W, slightly round plaques with diameters <1 mm were produced, indicating the presence of virions released from lysed cells (Figure 1a). Additionally, TEM was used to characterize the morphology of R8W; these analyses revealed that R8W has an equidistant and icosahedral head (65 ± 1 nm in diameter) with a short tail (12 ± 2 nm in length) (Figure 1b).

We determined the lytic cycle of R8W with a one-step growth curve at 0.01 multiplicity of infection. This growth curve showed a latent period of approximately 30 min, followed by a rise period of 45 min and a burst size of 88 plaque-forming units/cell (Figure 1c). These findings indicated that the burst size and rise period of R8W differed from those of previously described alterophages [15,16,19]. For example, alterophages AltAD45-P1 and P2 have a rise period of approximately 5 h [19].

The ability of R8W to cross-infect other *Alteromonas* spp. was investigated. The tested strains included a mostly complete collection of known *Alteromonas* type strains and a considerable number of *A. macleodii* isolates. We used various phage abundances (10^5^, 10^7^, 10^9^, and 10^11^ plaque-forming units/mL) to determine the efficiency of infection. To the best of our knowledge, R8W exhibits the broadest host range among all known alterophages. Previous studies have shown that alterophages generally exhibit a narrow host range. For example, two siphophages (JH01 and PB15) and one myophage (vB_AmeM_PT11-V22) only infected their original hosts [14,16,18]. Furthermore, four N4-like podophages (vB_AmaP_AltAD45-P1, P2, P3, and P4) were able to infect two *Alteromonas, A. mediterranea* and *A. macleodii* [19]. In our study, R8W could infect 35 of the 79 strains tested (infection rate of 44.30%); these 35 strains included nine species (Table 2).

According to their isolation sources, the *Alteromonas* spp. in this study were divided into five categories, including surface seawaters, subsurface seawaters, deep seawaters, sediment, and other origins. The respective infection rates were 46%, 44%, 50%, 36%, and 37.5%. Therefore, R8W exhibited particularly strong specificity for strains isolated from deep seawaters (Table 2). Additionally, the 35 tested strains that could be infected by R8W had diverse global geographical origins (Table 2). The rate of R8W infection among *Alteromonas* spp. isolated from surface and subsurface seawaters was nearly 50%. Overall, these results suggest that R8W can survive in multiple oceanic regions and might be widely distributed in the global ocean.

### 3.2. Genomic Characteristics

**Figure 2:**
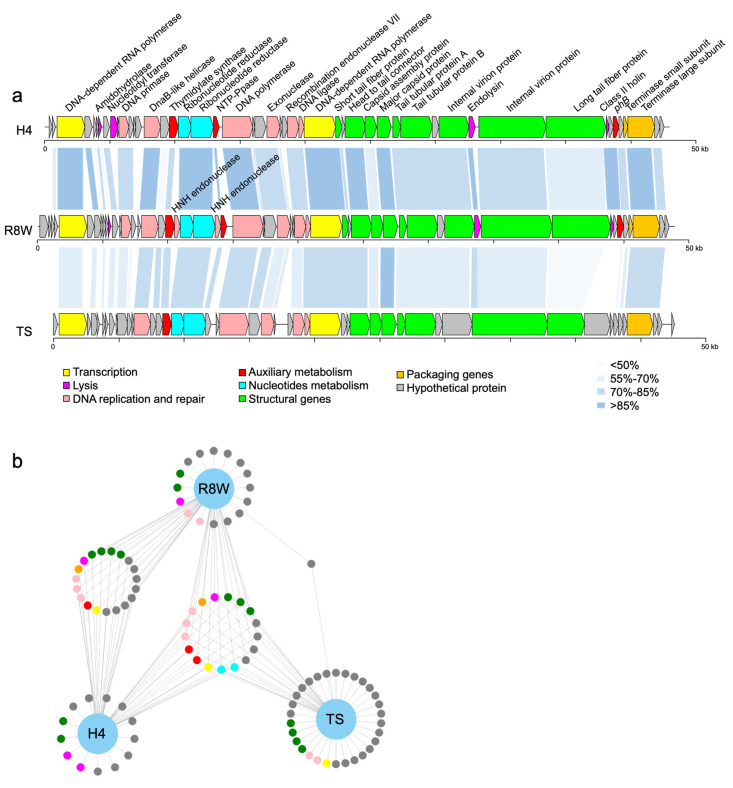
(**a**) Genome organization and comparison of the phage R8W to vB_AspP-H4/4 (H4) (GenBank accession no. MF278336) and prokaryotic dsDNA virus TS (GenBank accession no. MK892710, isolated from the Tara Oceans expedition Tp1_25_SUR_0-0d2_C3569776_1). Arrows indicate the direction of transcription of each gene. Each color indicates a putative function. The color gradients represent the amino acid sequence identity obtained from BLASTP matching. (**b**) The network diagram shows the similarity between three phage genomes. The nodes represent genes, and the other end is a phage connected by lines. Genes from different phages that are in the same loop indicate that the similarity between the two genes is greater than 70%.

Analysis of the R8W genome revealed 48,825 bp of double-stranded DNA with a G+C content of 40.55%. R8W has a linear genome with direct terminal repeats of 202 bp (Supplementary Data 1). R8W belongs to the T7-like phages, in which the direct terminal repeats are recognized by the terminase as a fixed site and generated by DNA replication during packaging [28]. The R8W genome has 53 putative protein-coding sequences, which comprise 96.95% of the genome, with amino acid sequence lengths of 35 to 1818. Of those putative protein-coding sequences, 24 gene products were identified as hypothetical proteins, while 29 gene products were arranged in eight functional categories (Figure 2a and Supplementary Materials Table S2), including auxiliary metabolism, DNA interactions, signal transduction regulation, and packaging. Among the genomic loci, predicted DNA replication and repair proteins occupied the largest proportion (31.24%). No tRNA sequences were identified in the R8W genome using the tRNAscan-SE program, suggesting that there may be close interactions between the phage and its host during protein synthesis [48].

**Figure 3:**
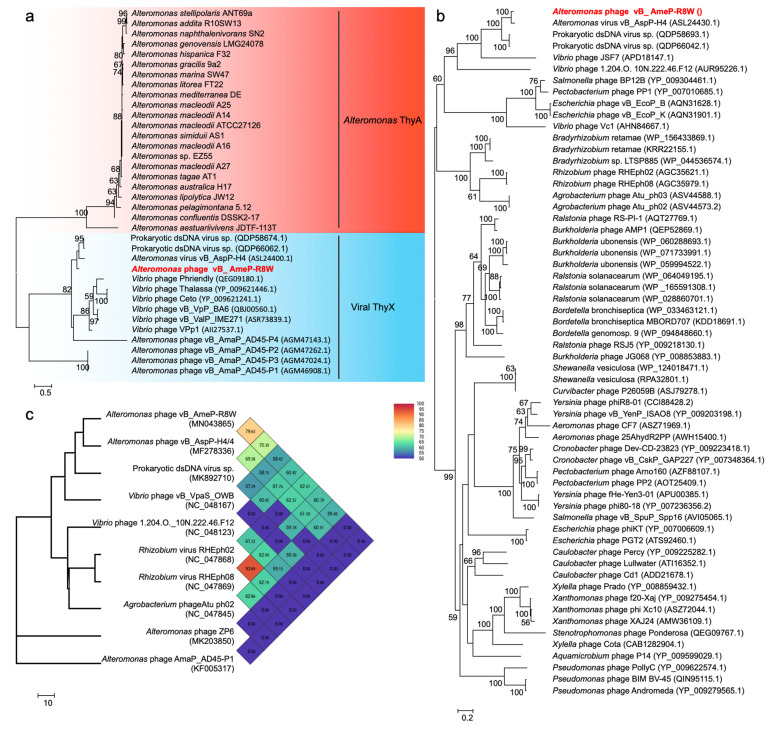
(**a**) Phylogenetic analysis of thymidylate synthase amino acid sequences of 22 *Alteromonas* and 14 phages. (**b**) Phylogenetic analysis of TerL amino acid sequences of 60 phages. (**c**) The genome-wide tree based on the average nucleotide identity (ANI) from 10 phages. Numbers at the nodes indicate bootstrap values (1000 replications and values >50%).

Phage-encoded AMGs can directly modulate host function and enhance viral propagation, subsequently altering biogeochemical cycling. AMGs have been widely reported in cyanophages, where they are involved in photosynthesis and nitrogen metabolism [3,4,5]. In this study, we identified three putative AMGs in the R8W genome: *g19*, *g24*, and *g48*. *g19* encodes ThyX, a thymidylate synthase with two forms, ThyX and ThyA, which are primarily found in phages and bacteria, respectively. Thymidylate synthase can catalyze the synthesis of thymidylate and bind RNA to inactivate regulatory gene expression [49,50]. We built a phylogenetic tree of thymidylate synthase based on amino acid sequences in which the known proteins were divided into two phylogenetic groups: viral ThyX and *Alteromonas* ThyA (Figure 3a). *g24* encodes a nucleoside triphosphate pyrophosphohydrolase, which exhibits robust similarity to dUTP pyrophosphatase and nucleoside triphosphate pyrophosphohydrolase MazG proteins; however, it only consists of a single MazG-like domain, which may influence the host signal and survival rate during phage reproduction [51]. These two genes were previously found in other phages, including *Vibrio* phages, *Roseobacter* phages, and cyanophages [40,52]. G48 is annotated as the putative DNA-binding response regulator, PhoB, which functions as a two-component response regulator [53,54]. Phosphorylation of PhoB can enhance the affinity of DNA binding to regulate transcription in low-phosphate environments [53,54]. The presence of *phoB* gene in the R8W genome implies potential adaptations to low-phosphate environments. These three AMGs were associated with viral nucleotide metabolism and host metabolism, potentially improving DNA replication efficiency by enabling R8W to utilize its own genes to acquire the metabolites needed during phage infection.

Comparative genomic analysis revealed that R8W has isomorphic modules and gene synteny similar to those of alterophage vB_AspP-H4/4 (H4) and a prokaryotic virus in a metagenome-assembled bin (TS) (Figure 2a). Phage H4 (GenBank accession no. MF278336) was isolated from North Sea water using *Alteromonas addita* as its host [17]. TS (GenBank accession no. MK892710) was assembled from viral metagenome sequences from the Tara Oceans expedition at station TARA_022 (latitude *N* = 39.8386, longitude *E* = 17.4155, depth = 5 m) [5]. Genome maps showed that structural proteins are concentrated in the middle and right-hand regions of R8W, while genes for DNA replication and repair are located upstream of the structural genes. Furthermore, nucleotide metabolism genes and AMGs are distributed among the DNA replication and repair genes. Based on the total amino acid matches, R8W has remarkable similarity with H4 and TS (29.56–98.55% and 38.67–85.99% identities, respectively). In particular, nucleotide metabolism genes, packaging genes, and AMGs were common among the three phages (identity > 70%) (Figure 2b, Supplementary Materials Table S3); these gene products are important for ensuring full phage assembly and host interactions [23,40,55].

### 3.3. Broad Host Range of Phage R8W

Adsorption of phages to their hosts is the first step in the lytic cycle [56]. Phages have been found to encode RBPs, which are a critical structure in host-specific recognition [14,56]. RBPs are usually located at the distal end of the tail along with tail fiber proteins, tail spike proteins, and tail tip proteins [14,56]. Including R8W, all the 11 known alterophages can infect 10 different *Alteromonas* species [14,15,16,17,18,19]. Our analysis demonstrated 13 types of RBPs in these alterophages (Table 3), including pectin degradation protein, glycoside hydrolase, endo-*N*-acetyl neuraminidase, glucosidase, xylosidase, and glucanase. They may specifically recognize host flagellum glycosylation, capsular exopolysaccharide, and lipopolysaccharides [22]. These genes were potential phages receptors [22]. In a previous study, *Klebsiella* phages were found to exhibit 11 types of RBPs with depolymerase activity, which enabled infection of distinct *Klebsiella pneumoniae* strains with thick polysaccharide capsules [33]. The *Escherichia coli* phage phi92 contains five types of RBPs, two of which have degrading enzyme activity (endo-*N*-acyl neuraminidase and colanidase). This phage can infect eight *E. coli* strains and 19 *Salmonella* strains, thus demonstrating a broad host range [57].

**Figure 4:**
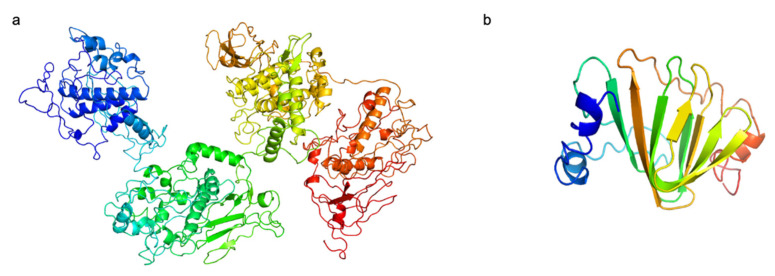
The predicted structures of putative receptor binding proteins in vB_AmeP-R8W (R8W). (**a**) Gp45, putative long tail fibers. (**b**) Gp34, putative short tail fibers. N-terminal is colored blue, and C-terminal is colored red. The C-score of two proterins are −1.85 and −0.86, respectively.

Two putative RBPs were identified in the R8W genome, including Gp34 and Gp45; their predicted structures are shown in Figure 4. First, their functions were annotated as putative tail fibers. The N-terminal domain of Gp45 has a high degree of identity to the T7-tail fiber protein, as indicated by Pfam (bit score, 30.5; e-value, 2.5 × 10^−7^). T7-tail fiber proteins recognize bacterial outer membrane lipopolysaccharide [58,59]. Second, the C-terminal domain of Gp45 was predicted to be a glycoside hydrolase by HHpred (probability, 96.68%; e-value, 0.062; aligned cols, 171) and Phyre2 (confidence, 91.3%; identity, 27%); the C-terminal domain of Gp34 was predicted to be a dimethylsulfoniopropionate lyase by HHpred (probability, 99.11; e-value, 5.3 × 10^−9^; aligned cols, 94) and Phyer2 (confidence, 92.8%; identity, 18%). These results suggest that Gp34 and Gp45 may have depolymerization activity that enables hydrolysis of bacterial exopolysaccharide, which may be the first step in the R8W infection process. Indeed, *Alteromonas gracilis* 9a2, an exopolysaccharide producer, is efficiently infected by R8W. The exopolysaccharide of *A. gracilis* 9a2 consists of mannose, galactose, and glucose, which may be one of the R8W receptors, and be recognized by glycosyl hydrolase in R8W, thus facilitating R8W infection [60]. Thus, we speculate that its possession of two types of RBPs with depolymerization activity might contribute to the broader host range of R8W. The RBPs of the alterophages JH01 and vB_AmeM_PT11-V22 are capsid fiber protein and tail fiber protein, respectively. Additionally, the alterophages JH01 and vB_AmeM_PT11-V22 are only able to infect their original hosts, *A. marina* SW-47 and *A. mediterranea* PT11, respectively. [14,18]. Alterophage PB15 contains two RBPs and may infect a wide range of hosts, although the original study showed a narrow host range among the few host strains tested [16].

The host range of R8W may also be determined by its lytic ability to exit the host [56]. In the R8W genome, *g43* and *g46* are predicted to be putative endolysin and class II holin genes, respectively. These two gene products enable phages to initiate lysis at a specific point during the infection [56,61]. Holin protein can insert into the host cytoplasmic membrane, oligomerize, and form holes in the membrane [56,61]. Then, the endolysin passes through these holes to selectively degrade peptidoglycan [56,61]. The presence or absence of a holin gene in *Lactococcus lactis* phages affects their lytic efficiency [62]. The holin–endolysin system has also been found in alterophages vB_AcoS-R7M, ZP6 and H4. Thus, we infer that vB_AcoS-R7M, ZP6 and H4 have broad host ranges.

### 3.4. R8W Is Characterized as a New Species of Foturvirus Genus within the Autographiviridae Family

To evaluate the genetic relationships of R8W, we constructed a phylogenetic tree using 60 similar amino acid sequences of TerL (Figure 3b). R8W and H4 were grouped together into a clade containing two prokaryotic viruses in metagenome-assembled bins (TS and TS1) and two *Vibrio* phages, suggesting that these phages are close relatives. TS1 (GenBank accession no. MK892670) was assembled from viral metagenome sequences collected during the Tara Oceans expedition at station TARA_052 (latitude *N* = −16.957, longitude *E* = 53.9801). R8W and its closest relative H4 exhibited distinct genomic information and lower ANI values (69.38–79.63%) (Figure 3c); these findings implied that R8W is a novel alterophage species. BLASTN analysis also showed that the R8W genome was closely related to the H4 and TS genomes with sequence identities of 79.16% and 78.64%, respectively.

**Figure 5:**
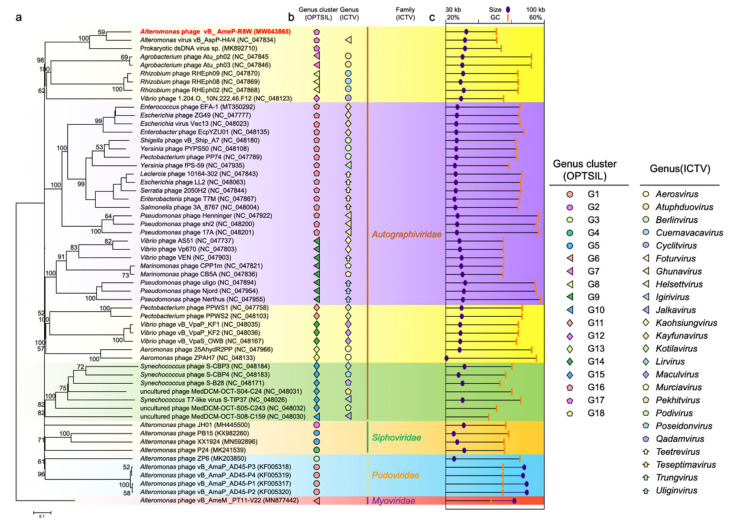
(**a**) Genome BLAST distance phylogeny (GBDP) tree of 57 virus genomes. Based on nucleotide sequences, the GBDP tree is reconstructed by VICTOR, which used the D6 formula and yielded an average support of 71%. Numbers at the nodes are GBDP pseudo-bootstrap values (100 replications and values >50%). (**b**) ICTV and OPTSIL clusters at the genus and family levels. Each genus is indicated by a unique shape and color. Background colors indicate the 8 OPTSIL clusters at the family level. (**c**) G + C content and genome sizes.

Phages are regarded as members of the same genus by the International Committee on Taxonomy of Viruses when their nucleotide sequence identities are greater than 50% [63]. The ANI values were between 69.38% and 79.63% for comparisons of R8W, H4, and TS (Figure 3c), suggesting that these phages can be grouped into a single genus but should be regarded as different species [40,63]. However, the application of ANI to taxonomy is limited to sequences with high coverage at the genome-wide level. We selected the 45 genomes of *Autographiviridae* viruses, 12 alterophages, and TS to reconstruct the genome BLAST distance phylogeny tree (Figure 5a). OPTSIL clustering produced 48 species clusters, 17 genera clusters, and 8 family clusters (Figure 5a,b). The tree showed reliable bootstrap values at most nodes. Both genome sizes and G + C contents of distinct species were strongly correlated by means of phylogenetic clustering (Figure 5a,c). The results of phylogenetic clustering were also consistent with the classifications defined by the International Committee on Taxonomy of Viruses (Figure 5a,b). The phylogenetic trees implied that R8W is closely related to *Vibrio* and *Rhizobium* phages; this relationship is also supported by phylogenetic analysis of the core protein, TerL (Figure 3c). The above results indicate that R8W is appropriately classified as a new species; moreover, R8W, H4, and TS belong to the *Foturvirus* genus in the *Autographiviridae* family.

## 4. Conclusions

In this study, we isolated and defined the novel alterophage species vB_AmeP-R8W using a deep-clade *Alteromonas mediterranea* as its host. This novel alterophage species belongs to the *Autographiviridae* family and thus extends our knowledge of alterophage–host interactions. This large survey concerning alterophage host range showed that R8W has a broad host range and has particularly strong specificity for *Alteromonas* strains isolated from deep waters. Numerous important functional proteins were found in R8W, such as RBPs, which potentially increase its replication success and host recognition. R8W possesses specific RBPs and a compatible holin–endolysin system to expand its host range. However, there were some limitations in terms of the proteomic analysis and structural resolution of R8W, which limited the ability to verify alterophage–*Alteromonas* interactions. Considering the importance of *Alteromonas* and the influences of alterophages on their hosts, more alterophages must be isolated to analyze their genomic and evolutionary diversities. In the future, more alterophage–*Alteromonas* model systems could be investigated, which will greatly enhance the overall knowledge regarding the ecological implications of alterophages and their hosts.

## Figures and Tables

**Table 1:** Statistics of reported genomic information of alterophages.

Phage Isolate	Isolation Host	Family	Size (bp)	Number of Genes	GC (%)	GenBank
vB_AmeP-R8W	*A. mediterranea* DE	*Autographiviridae*	48,825	55	40.6	MW043865
vB_AspP-H4/4	*A. addita* H4	*Autographiviridae*	47,631	49	40.8	MF278336
vB_AmaP_AD45-P1	*A. macleodii* AD45	*Podoviridae*	103,910	129	43.2	KF005317
vB_AmaP_AD45-P2	*A. macleodii* AD45	*Podoviridae*	104,036	129	43.2	KF005320
vB_AmaP_AD45-P3	*A. macleodii* AD45	*Podoviridae*	101,724	124	43.2	KF005318
vB_AmaP_AD45-P4	*A. macleodii* AD45	*Podoviridae*	100,619	122	43.2	KF005319
ZP6	*A. macleodii* sp.	*Podoviridae*	37,743	46	50.1	MK203850
PB15	*A. gracilis* B15	*Siphoviridae*	37,333	61	45.5	KX982260
JH01	*A. marina* SW-47	*Siphoviridae*	46,500	58	44.4	MH445500
P24	*A. macleodii* sp.	*Siphoviridae*	46,945	74	43.8	MK241539
XX1924	*A. litorea* TF-22	*Siphoviridae*	40,580	64	43.7	MN592896
vB_AcoS-R7M	*A. confluentis* DSSK2-12	*Siphoviridae*	56,163	67	45.6	MT345684
vB_AmeM_PT11-V22	*A. mediterranea* PT11	*Myoviridae*	92,760	156	38.4	MN877442

**Table 2:** The host range of vB_AmeP-R8W (R8W).

Strain	Isolated From *	Depth	PFU/mL			
			10^11^	10^9^	10^7^	10^5^
*Alteromonas macleodii* ATCC 27126^T^	Hawaii, Pacific Ocean Oahu	Surface seawaters	+	+	+	+
*Alteromonas marina* SW-47^T^	Eastern Sea, Korea	Surface seawaters	+	+	+	+
*Alteromonas stellipolaris* ANT 69a^T^	Antarctica	Surface seawaters	+	+	+	+
*Alteromonas macleodii* AD45	Mediterranean Sea	Surface seawaters	+	+	+	+
*Alteromonas addita* R10SW13^T^	Chazhma Bay, Sea of Japan, Pacific Ocean	Surface seawaters	+	+	+	+
*Alteromonas macleodii* MCCC 1K00172	South China Sea	Surface seawaters	+	+	−	−
*Alteromonas macleodii* MCCC 1K00560	Eastern Pacific Ocean	Surface seawaters	+	+	−	−
*Alteromonas macleodii* AD037	Port Dickson, Malaysia	Surface seawaters	+	+	−	−
*Alteromonas macleodii* BSH94-8	Black Sea Karadag	Surface seawaters	+	+	−	−
*Alteromonas macleodii* AD006	Port Dickson, Malaysia	Surface seawaters	+	+	−	−
*Alteromonas macleodii* MCCC 1K01332	East Pacific Ocean	Surface seawaters	+	−	−	−
*Alteromonas confluentis* DSSK2-12^T^	Jeju Island, South Korea	Surface seawaters	+	−	−	−
*Alteromonas mediterranea* EC615	English Channel	Surface seawaters	+	−	−	−
*Alteromonas macleodii* MCCC 1K00460	Western Pacific Ocean	Surface seawaters	−	−	−	−
*Alteromonas macleodii* MCCC 1K01839	Western Pacific Ocean	Surface seawaters	−	−	−	−
*Alteromonas macleodii* MCCC 1K01358	Eastern Pacific Ocean	Surface seawaters	−	−	−	−
*Alteromonas macleodii* MCCC 1K00811	South China Sea	Surface seawaters	−	−	−	−
*Alteromonas australica* H 17^T^	Port Phillip Bay, Tasman Sea, Pacific Ocean	Surface seawaters	−	−	−	−
*Alteromonas tagae* AT1^T^	Er-Jen River estuary, Tainan	Surface estuarine waters	−	−	−	−
*Alteromonas lipolytica* JW12^T^	Arabian Sea, Indian Ocean	Surface seawaters	−	−	−	−
*Alteromonas macleodii* BS11	Black Sea Karadag	Surface seawaters	−	−	−	−
*Alteromonas mediterranea* MED64	Aegean Sea, Mediterranean	Surface seawaters	−	−	−	−
*Alteromonas macleodii* BS7	Black Sea Karadag	Surface seawaters	−	−	−	−
*Alteromonas macleodii* EC673	English Channel	Surface seawaters	−	−	−	−
*Alteromonas alba* 190^T^	Western Pacific Ocean	Surface seawaters	−	−	−	−
*Alteromonas macleodii* BSH84-3	Black Sea Karadag	Surface seawaters	−	−	−	−
*Alteromonas macleodii* BS8	Black Sea Karadag	Surface seawaters	−	−	−	−
*Alteromonas simiduii* AS1^T^	Er-Jen River estuary, Tainan	Surface estuarine waters	−	−	−	−
*Alteromonas macleodii* MCCC 1K01842	Western Pacific Ocean	Subsurface seawaters (75 m)	+	+	−	−
*Alteromonas macleodii* MCCC 1K01832	Western Pacific Ocean	Subsurface seawaters (30 m)	+	−	−	−
*Alteromonas macleodii* MCCC 1K01840	Western Pacific Ocean	Subsurface seawater (30 m)	+	−	−	−
*Alteromonas macleodii* MCCC 1K01294	Western Pacific Ocean	Subsurface seawaters (75 m)	+	−	−	−
*Alteromonas macleodii* MCCC 1K01823	Western Pacific Ocean	Subsurface seawaters (75 m)	−	−	−	−
*Alteromonas macleodii* A16(2794)	South China Sea	Subsurface seawater (75 m)	−	−	−	−
*Alteromonas macleodii* A14(2783)	South China Sea	Subsurface seawaters (75 m)	−	−	−	−
*Alteromonas macleodii* MCCC 1K01274	Western Pacific Ocean	Subsurface seawaters (100 m)	−	−	−	−
*Alteromonas macleodii* MCCC 1K01826	Western Pacific Ocean	Subsurface seawaters (100 m)	−	−	−	−
*Alteromonas mediterranea* DE^T^	Adriatic Sea, Urania Basin	Deep seawaters (1000 m)	+	+	+	+
*Alteromonas macleodii* MCCC 1A04487	Northwestern Pacific Ocean	Deep seawaters (2700 m)	+	+	+	−
*Alteromonas mediterranea* DE1	Adriatic Sea, Urania Basin	Deep seawaters (1000 m)	+	+	−	−
*Alteromonas macleodii* MCCC 1A07993	Southern Atlantic Ocean	Deep seawaters (2147 m)	+	+	−	−
*Alteromonas macleodii* MCCC 1A09262	Southern Atlantic Ocean	Deep seawaters (3047 m)	+	+	−	−
*Alteromonas mediterranea* UM7	Ionian Sea, Uranian Basin Western of Crete	Deep seawaters (3475 m)	+	+	−	−
*Alteromonas mediterranea* UM8	Ionian Sea, Uranian Basin Western of Crete	Deep seawaters (3475 m)	+	+	−	−
*Alteromonas macleodii* MCCC 1A00323	Atlantic Ocean	Deep seawaters (3542 m)	+	+	−	−
*Alteromonas macleodii* MCCC 1K02087	South China Sea	Deep seawaters (1700 m)	+	−	−	−
*Alteromonas macleodii* MCCC 1K00565	Eastern Pacific Ocean	Deep seawaters (5098 m)	+	−	−	−
*Alteromonas macleodii* MCCC 1K00800	Eastern Pacific Ocean	Deep seawaters (1000 m)	−	−	−	−
*Alteromonas macleodii* MCCC 1A02046	Indian Ocean	Deep seawaters (2391 m)	−	−	−	−
*Alteromonas mediterranea* UM4b	Ionian Sea, Uranian Basin Western of Crete	Deep seawaters (3455 m)	−	−	−	−
*Alteromonas mediterranea* U4	Ionian Sea, Uranian Basin Western of Crete	Deep seawaters (3475 m)	−	−	−	−
*Alteromonas mediterranea* U7	Ionian Sea, Uranian Basin Western of Crete	Deep seawaters (3500 m)	−	−	−	−
*Alteromonas mediterranea* U8	Ionian Sea, Uranian Basin Western of Crete	Deep seawaters (3500 m)	−	−	−	−
*Alteromonas macleodii* U12	Ionian Sea, Uranian Basin Western of Crete	Deep seawaters (3500 m)	−	−	−	−
*Alteromonas macleodii* A25	South China Sea	Deep seawaters (4058 m)	−	−	−	−
*Alteromonas macleodii* A27	South China Sea	Deep seawaters (4058 m)	−	−	−	−
*Alteromonas* sp. MCCC 1A07988	Southern Atlantic Ocean	Deep seawaters (5610 m)	−	−	−	−
*Alteromonas gracilis* 9a2^T^	Pacific Ocean	Sediment (6310 m)	+	+	+	+
*Alteromonas* sp. MCCC 1A09157	Southern Atlantic Ocean	Sediment	+	+	−	−
*Alteromonas naphthalenivorans* SN2^T^	Taean, South Korea	Sediment (tidal-flat)	+	+	−	−
*Alteromonas macleodii* MCCC 1K02779	Atlantic Ocean	Sediment (2577 m)	+	−	−	−
*Alteromonas* sp. MCCC 1A09130	Southern Atlantic Ocean	Sediment	+	−	−	−
*Alteromonas macleodii* MCCC 1K02456	Northwestern Indian Ocean	Sediment (1818 m)	−	−	−	−
*Alteromonas macleodii* MCCC 1K02451	Northwestern Indian Ocean	Sediment (2009 m)	−	−	−	−
*Alteromonas* sp. MCCC 1A08050	Southern Atlantic Ocean	Sediment (2481 m)	−	−	−	−
*Alteromonas macleodii* MCCC 1K02444	Northwestern Indian Ocean	Sediment (2540 m)	−	−	−	−
*Alteromonas pelagimontana* 5.12^T^	Indian Ocean	Sediment (2681 m)	−	−	−	−
*Alteromonas macleodii* MCCC 1K01703	Atlantic Ocean	Sediment (2781m)	−	−	−	−
*Alteromonas macleodii* MCCC 1K01716	Atlantic Ocean	Sediment (2781 m)	−	−	−	−
*Alteromonas litorea* TF-22^T^	Korea, Yellow Sea	Sediment (Intertidal)	−	−	−	−
*Alteromonas aestuariivivens* JDTF-113	Jindo, South Korea	Sediment (tidal-flat)	−	−	−	−
*Alteromonas* sp. EZ55	Tropical Pacific Ocean	*Prochlorococcus* culture (20 m)	+	+	+	+
*Alteromonas macleodii* MCCC 1F01223	Xiamen, China	Algae culture	+	+	−	−
*Alteromonas hispanica* F-32^T^	Fuente de Piedra, southern Spain	Hypersaline water	+	+	−	−
*Alteromonas genovensis* LMG 24078^T^	Genoa, Italy	Biofilm	−	−	−	−
*Alteromonas macleodii* MCCC 1K02452	Northwestern Indian Ocean	Olivine (3042 m)	−	−	−	−
*Alteromonas macleodii* MCCC 1K00767	Eastern Pacific Ocean	Seawaters (500 m)	−	−	−	−
*Alteromonas macleodii* MCCC 1K01276	Western Pacific Ocean	Seawaters (300 m)	−	−	−	−
*Alteromonas macleodii* AS7	Andaman Sea	NA	−	−	−	−

* The strains isolated from the same sea area may be isolated from different stations. Please refer to the attachment for Supplementary Materials Table S1.

**Table 3:** RBPs of alterophages.

Phage	#Accession	aa Length	NCBI Annotation	HHpred (Probability|E-Value|Aligned Cols|Identities)	Phyre2 (Confidence|Identity)	β-Helix
vB_AmeP-R8W	-	156	tail fiber protein	pectin degradation protein (99.11%|5.3 × 10^−9^|94|13%)	dimethylsulfoniopropionate lyase (92.8%|18%)	YES
vB_AmeP-R8W	-	1492	tail fiber protein	glycoside hydrolase (96.68%|0.062|171|18%)	hydrolase,sialidase (91.3%|27%)	YES
vB_AspP-H4/4	ASL24413	157	tail fiber protein	pectin degradation protein (99.12%|4.6 × 10^−9^|83|19%)	dimethylsulfoniopropionate lyase (92.9%|18%)	YES
vB_AspP-H4/4	ASL24424	1524	tail fiber protein	glycoside hydrolase (97.14%|0.021|187|19%)	hydrolase,sialidase (91.3%|27%)	YES
Prokaryotic dsDNA virus TS	AWN07083	777	tail fiber protein	pectin degradation protein (99.23%|7.7 × 10^−10^|83|18%)	dimethylsulfoniopropionate lyase (92.3%|18%)	YES
Prokaryotic dsDNA virus TS	QDP58699	954	tail fiber protein	tail fiber protein (99.82%|6.6 × 10^−18^|278|14%)	tailspike gp27 (60.5%|23%)	YES
ZP6	AZS06567	873	tailspike protein	phiAB6 tailspike (99.92%|3.5 × 10^−22^|402|13%)	alpha-1,3-glucanase (99.95%|14%)	YES
ZP6	QMS42070	629	tailspike protein	tailspike protein (99.92%|4.8 × 10^−22^|383|14%)	glucan 1,3-beta-glucosidase (96.9%|26%)	YES
vB_AmaP_AD45-P1	AGM46838	1545	tail fiber protein	endo-*N*-acetylneuraminidase (98.09%|0.000014|95|21%)	chaperone,endo-*N*-acetylneuraminidase (99.1%|18%)	YES
vB_AmaP_AD45-P1	AGM46839	1236	tail fiber protein	NF	long-tail fiber (84.8%|11%)	YES
vB_AmaP_AD45-P2	AGM47190	1545	tail fiber protein	endo-*N*-acetylneuraminidase (98.09%|0.000014|95|21%)	chaperone,endo-*N*-acetylneuraminidase (99.1%|18%)	YES
vB_AmaP_AD45-P2	AGM47191	1236	tail fiber protein	NF	long-tail fiber (84.8%|11%)	YES
vB_AmaP_AD45-P3	AGM46957	1545	tail fiber protein	endo-*N*-acetylneuraminidase (97.14%|0.000014|57|18%)	chaperone,endo-*N*-acetylneuraminidase (99.1%|18%)	YES
vB_AmaP_AD45-P4	AGM46958	1236	tail fiber protein	NF	long-tail fiber (84.8%|11%)	YES
vB_AmaP_AD45-P4	AGM47074	1545	tail fiber protein	endo-*N-*acetylneuraminidase (98.09%|0.000014|95|21%)	chaperone,endo-*N*-acetylneuraminidase (99.1%|18%)	YES
vB_AmaP_AD45-P4	AGM47075	1236	tail fiber protein	NF	long-tail fiber (84.8%|11%)	YES
PB15	APC46581	748	tailspike protein	particle-associated lyase (99.86%|3.1 × 10^−18^|328|14%)	hydrolase, xylosidase (83.9%|25%)	YES
PB15	APC46582	731	tail fiber protein	endo-beta-*N-*acetylglucosaminidase (97.08%| 0.019|119|5%)	hydrolase, spgh29 (98.4%|18%)	YES
JH01	AWY02808	112	capsid fiber protein	Capsid fiber protein (64.4|24|238|347) alpha-galactosidase (99.35%|5.4 × 10^−11^|93|14%)	altronate hydrolase (53.4%|20%)	NO
XX1924	QGZ13097	518	discoidin domain-containing protein	endo-beta-*N*-acetylglucosaminidase (95.448%| 0.016|119|5%)	hydrolase, spgh29 (96.5%|18%)	YES
XX1924	QGZ13160	352	tail fiber protein	tailspike protein (97.81%|0.0075|212|11%)	antimicrobial protein, neutrophil defensin 4 (51.6%|62%)	NO
P24	AZU97343	361	tail fiber protein	NF	nf	
vB_AcoS-R7M	YP_009859590	273	ribonuclease III	NF	sugar binding protein (47%|10%)	NO
vB_AmeM_PT11-V22	QHZ59724	369	tail fiber protein	-	-	-

NF = not found.

## Data Availability

The complete genome sequence of alterophage vB_AmeP-R8W was submitted to the GenBank database under accession number MW043865.

## Supplementary Materials

The following are available online at https://www.mdpi.com/article/10.3390/v13060987/s1, Table S1. Genomic information of *Alteromonas* strains used in this study; Table S2. vB_AmeP-R8W (R8W) gemone annotation; Table S3. The genetic comparison of the phage vB_AmeP-R8W (R8W), vB_AspP-H4/4 (H4) and prokaryotic dsDNA virus (TS) (similarity > 70%); Figure S1. Station map indicating location where vB_AmeP-R8W (R8W) was isolated. Supplementary Data 1. Results of R8W PhageTerm analysis.
