# Supplementary material for: A Novel Broad Host Range Phage Infecting Alteromonas
Source: Viruses. 2021 May 26;13(6):987. doi: 10.3390/v13060987 (PMC8228385; doi:10.3390/v13060987)
Supplement: Supplementary file 1 [file viruses-13-00987-s001.zip › viruses-1174591-supplementary.pdf]

Supplementary Materials

Table S1. Genomic information of *Alteromonas* strains used in this study

Strain	#Accession	Isolated from	Depth	Reference
Alteromonas macleodii ATCC 27126^{T}	NA	Hawaii, Pacific Ocean Oahu	Surface seawaters	[1]
Alteromonas marina SW-47^{T}	NA	Eastern Sea, Korea	Surface seawaters	[2]
Alteromonas stellipolaris ANT 69a^{T}	CP013926	Antarctica	Surface seawaters	[3]
Alteromonas macleodii AD45	CP003874	Mediterranean Sea	Surface seawaters	[4]
Alteromonas tagae AT1^{T}	NA	Er-Jen River estuary, Tainan	Surface estuarine waters	[5]
Alteromonas macleodii MCCC 1K00172	NA	South China Sea	Surface seawaters	Unpublished[a]
Alteromonas macleodii MCCC 1K00560	NA	Eastern Pacific Ocean	Surface seawaters	Unpublished[a]
Alteromonas macleodii AD037	NZ_JWLY01000001.1	Port Dickson, Malaysia	Surface seawaters	Unpublished[b]
Alteromonas macleodii BSH94-8	NA	Black Sea Karadag	Surface seawaters	[6]
Alteromonas macleodii AD006	NZ_JWLX01000001.1	Port Dickson, Malaysia	Surface seawaters	[6]
Alteromonas macleodii MCCC 1K01332	NA	East Pacific Ocean	Surface seawaters	Unpublished[a]
Alteromonas confluentis DSSK2-12^{T}	NZ_MDHN01000001.1	Jeju Island, South Korea	Surface seawaters	[7]
Alteromonas mediterranea EC615	CP004846	English Channel	Surface seawaters	[8]
Alteromonas macleodii MCCC 1K00460	NA	Western Pacific Ocean	Surface seawaters	Unpublished[a]
Alteromonas macleodii MCCC 1K01839	NA	Western Pacific Ocean	Surface seawaters	Unpublished[a]

Table S1 | Continued

Strain	#Accession	Isolated from	Depth	Reference
Alteromonas macleodii MCCC 1K01358	NA	Eastern Pacific Ocean	Surface seawaters	Unpublished[a]
Alteromonas macleodii MCCC 1K00811	NA	South China Sea	Surface seawaters	Unpublished[a]
Alteromonas australica H 17[T]	CP008849	Port Phillip Bay, Tasman Sea, Pacific Ocean	Surface seawaters	[9]
Alteromonas addita R10SW13[T]	CP014322	Chazhma Bay, Sea of Japan, Pacific Ocean	Surface seawaters	[10]
Alteromonas lipolytica JW12[T]	NZ_MJIC01000001.1	Arabian Sea, Indian Ocean	Surface seawaters	[11]
Alteromonas macleodii BS11	CP003845	Black Sea Karadag	Surface seawaters	[8]
Alteromonas mediterranea MED64	CP004848	Aegean Sea, Mediterranean	Surface seawaters	[8]
Alteromonas macleodii BS7	NA	Black Sea Karadag	Surface seawaters	[8]
Alteromonas macleodii EC673	CP003844	English Channel	Surface seawaters	[8]
*Alteromonas alba*190[T]	NZ_PVNP01000003.1	Westernern Pacific	Surface seawaters	[12]
Alteromonas macleodii BSH84-3	NA	Black Sea Karadag	Surface seawaters	[6]
Alteromonas macleodii BS8	NA	Black Sea Karadag	Surface seawaters	[8]
Alteromonas simiduii AS1[T]	NA	Er-Jen River estuary, Tainan	Surface estuarine waters	[5]
Alteromonas macleodii MCCC 1K01842	NA	Western Pacific Ocean	Subsurface seawaters (75 m)	Unpublished[a]
Alteromonas macleodii MCCC 1K01832	NA	Western Pacific Ocean	Subsurface seawaters (30 m)	Unpublished[a]
Alteromonas macleodii MCCC 1K01840	NA	Western Pacific Ocean	Subsurface seawaters (30 m)	Unpublished[a]
Alteromonas macleodii MCCC 1K01294	NA	Western Pacific Ocean	Subsurface seawaters (75 m)	Unpublished[a]
Alteromonas macleodii MCCC 1K01823	NA	Western Pacific Ocean	Subsurface seawaters (75 m)	Unpublished[a]

Table S1 | Continued

Strain	#Accession	Isolated from	Depth	Reference
Alteromonas macleodii A16(2794)	NA	South China Sea	Subsurface seawaters (75 m)	Unpublished[c]
Alteromonas macleodii A14(2783)	NA	South China Sea	Subsurface seawaters (75 m)	Unpublished[c]
Alteromonas macleodii MCCC 1K01274	NA	Western Pacific Ocean	Subsurface seawaters (100 m)	Unpublished[a]
Alteromonas macleodii MCCC 1K01826	NA	Western Pacific Ocean	Subsurface seawaters (100 m)	Unpublished[a]
Alteromonas mediterranea DE^T	CP001103	Adriatic Sea, Urania Basin	Deep seawaters (1000 m)	[6,13]
Alteromonas macleodii MCCC 1A04487	NA	Northwestern Pacific Ocean	Deep seawaters (2700 m)	Unpublished[a]
Alteromonas mediterranea DE1	CP003917	Adriatic Sea, Urania Basin	Deep seawaters (1000 m)	[6]
Alteromonas macleodii MCCC 1A07993	NA	Southern Atlantic Ocean	Deep seawaters (2147 m)	Unpublished[a]
Alteromonas macleodii MCCC 1A09262	NA	Southern Atlantic Ocean	Deep seawaters (3047 m)	Unpublished[a]
Alteromonas mediterranea UM7	CP004853	Ionian Sea, Uranian Basin Western of Crete	Deep seawaters (3475 m)	[14]
Alteromonas mediterranea UM8	CP013928	Ionian Sea, Uranian Basin Western of Crete	Deep seawaters (3475 m)	[14]
Alteromonas macleodii MCCC 1A00323	NA	Atlantic Ocean	Deep seawaters (3542 m)	Unpublished[a]
Alteromonas macleodii MCCC 1K02087	NA	South China Sea	Deep seawaters (1700 m)	Unpublished[a]
Alteromonas macleodii MCCC 1K00565	NA	Eastern Pacific Ocean	Deep seawaters (5098 m)	Unpublished[a]
Alteromonas macleodii MCCC 1K00800	NA	Eastern Pacific Ocean	Deep seawaters (1000 m)	Unpublished[a]
Alteromonas macleodii MCCC 1A02046	NA	Indian Ocean	Deep seawaters (2391 m)	Unpublished[a]
Alteromonas mediterranea UM4b	CP004855	Ionian Sea, Uranian Basin Western of Crete	Deep seawaters (3455 m)	[14]
Alteromonas mediterranea U4	CP004849	Ionian Sea, Uranian Basin Western of Crete	Deep seawaters (3475 m)	[14]

Table S1 | Continued

Strain	#Accession	Isolated from	Depth	Reference
Alteromonas mediterranea U7	CP004851	Ionian Sea, Uranian Basin Western of Crete	Deep seawaters (3500 m)	[14]
Alteromonas mediterranea U8	CP004852	Ionian Sea, Uranian Basin Western of Crete	Deep seawaters (3500 m)	[14]
Alteromonas macleodii U12	NA	Ionian Sea, Uranian Basin Western of Crete	Deep seawaters (3500 m)	[14]
Alteromonas macleodii A25	NA	South China Sea	Deep seawaters (4058 m)	Unpublished[c]
Alteromonas macleodii A27	NA	South China Sea	Deep seawaters (4058 m)	Unpublished[c]
Alteromonas sp. MCCC 1A07988	NA	Southern Atlantic Ocean	Deep seawaters (5610 m)	Unpublished[a]
Alteromonas gracilis 9a2[T]	NZ_PVNO01000001.1	Pacific Ocean(mannose, galactose and glucose.)	Sediment (6310 m)	[15]
Alteromonas sp. MCCC 1A09157	NA	Southern Atlantic Ocean	Sediment	Unpublished[a]
Alteromonas naphthalenivorans SN2[T]	CP002339	Taean, South Korea	Sediment (tidal-flat)	[16]
Alteromonas macleodii MCCC 1K02779	NA	Atlantic Ocean	Sediment (2577 m)	Unpublished[a]
Alteromonas sp. MCCC 1A09130	NA	Southern Atlantic Ocean	Sediment	Unpublished[a]
Alteromonas macleodii MCCC 1K02456	NA	Northwestern Indian Ocean	Sediment (1818 m)	Unpublished[a]
Alteromonas macleodii MCCC 1K02451	NA	Northwestern Indian Ocean	Sediment (2009 m)	Unpublished[a]
Alteromonas sp. MCCC 1A08050	NA	Southern Atlantic Ocean	Sediment (2481 m)	Unpublished[a]
Alteromonas macleodii MCCC 1K02444	NA	Northwestern Indian Ocean	Sediment (2540 m)	Unpublished[a]
Alteromonas pelagimontana 5.12[T]	CP052766	Indian Ocean	Sediment (2681 m)	[17]
Alteromonas macleodii MCCC 1K01703	NA	Atlantic Ocean	Sediment (2781m)	Unpublished[a]
Alteromonas macleodii MCCC 1K01716	NA	Atlantic Ocean	Sediment (2781 m)	Unpublished[a]

Table S1 | Continued

Strain	#Accession	Isolated from	Depth	Reference
Alteromonas litorea TF-22^T	NA	Korea, Yellow Sea	Sediment (Intertidal)	[18]
Alteromonas aestuariivivens JDTF-113	NZ_QRHA01000001.1	Jindo, South Korea	Sediment (tidal-flat)	[19]
Alteromonas sp. EZ55	NZ_CABDXN010000001	Tropical Pacific Ocean	Prochlorococcus culture (20 m)	[20]
Alteromonas macleodii MCCC 1F01223	NA	Xiamen, China	Algae culture	Unpublished[a]
Alteromonas hispanica F-32^T	JAAAWP010000001.1	Fuente de Piedra, southern Spain	Hypersaline water	[21]
Alteromonas genovensis LMG 24078^T	NZ_JAAAWO010000001	Genoa, Italy	Biofilm	[22]
Alteromonas macleodii MCCC 1K02452	NA	Northwestern Indian Ocean	Olivine (3042 m)	Unpublished[a]
Alteromonas macleodii MCCC 1K00767	NA	Eastern Pacific Ocean	Seawaters (500 m)	Unpublished[a]
Alteromonas macleodii MCCC 1K01276	NA	Western Pacific Ocean	Seawaters (300 m)	Unpublished[a]
Alteromonas macleodii AS7	NA	Andaman Sea	NA	Unpublished[b]

a: Strains were purchased from the Marine Culture Collection of China (MCCC. China)

b: Strains were purchased from the German Collection of Microorganisms and Cell Cultures (DSMZ, Braunschweig, Germany)

c: Strains were isolated from our laboratory.

Table S2. vB_AmeP-R8W (R8W) genome annotation

Genes No.	Start	End	Length	Stand	GC%	Putative function	Functional categories
1	156	788	633	+	0.382	hypothetical protein	
2	802	969	168	+	0.363	hypothetical protein	
3	1020	1247	228	+	0.333	hypothetical protein	
4	1249	1512	264	+	0.337	hypothetical protein	
5	1608	3734	2127	+	0.414	DNA-dependent RNA polymerase	Transcription
6	3778	4320	543	+	0.418	hypothetical protein	
7	4335	4874	540	+	0.433	hypothetical protein	
8	4814	5011	198	+	0.449	hypothetical protein	
9	4998	5210	213	+	0.352	hypothetical protein	
10	5197	5376	180	+	0.433	hypothetical protein	
11	5373	5597	225	+	0.364	amidohydrolase	lysis
12	5711	6151	441	+	0.469	hypothetical protein	
13	6264	6434	171	+	0.427	hypothetical protein	
14	6379	7158	780	+	0.421	DNA primase	DNA replication and repair
15	7149	7514	366	+	0.393	hypothetical protein	
16	7644	7850	207	+	0.386	hypothetical protein	
17	7956	9203	1248	+	0.387	DnaB-like helicase	DNA replication and repair

Table S2 | Continued

Genes No.	Start	End	Length	Stand	GC%	Putative function	Functional categories
18	9203	9787	585	+	0.388	hypothetical protein	
19	9780	10451	672	+	0.409	thymidylate synthase	auxiliary metabolism
20	10459	10917	459	+	0.394	HNH endonuclease	DNA replication and repair
21	10920	11930	1011	+	0.394	ribonucleotide reductase, small chain	nucleotides metabolism
22	11906	13597	1692	+	0.418	ribonucleotide reductase, barrel domain	nucleotides metabolism
23	13588	14016	429	+	0.378	HNH endonuclease	DNA replication and repair
24	14009	14449	441	+	0.379	NTP-Ppase	auxiliary metabolism
25	14954	17266	2313	+	0.405	DNA polymerase	DNA replication and repair
26	17266	17394	129	+	0.349	hypothetical protein	
27	17394	18275	882	+	0.409	hypothetical protein	
28	18355	19353	999	+	0.395	5'-3' exonuclease	DNA replication and repair
29	19328	19546	219	+	0.397	hypothetical protein	
30	19536	19667	132	+	0.311	hypothetical protein	
31	19664	20539	876	+	0.4	DNA ligase	DNA replication and repair
32	20532	20918	393	+	0.413	recombination endonuclease VII	DNA replication and repair
33	20915	23305	2391	+	0.409	DNA-dependent RNA polymerase	Transcription
34	23332	23802	471	+	0.395	short tail fiber protein	structural genes
35	23778	24026	249	+	0.394	hypothetical protein	

Table S2 | Continued

Genes No.	Start	End	Length	Stand	GC%	Putative function	Functional categories
36	24035	25573	1539	+	0.406	head to tail connector	structural genes
37	25576	26445	870	+	0.413	capsid assembly protein	structural genes
38	26473	27576	1104	+	0.448	putative major capsid protein	structural genes
39	27687	28277	591	+	0.393	tail tubular protein A	structural genes
40	28286	30640	2355	+	0.403	tail tubular protein B	structural genes
41	30640	31182	543	+	0.411	hypothetical protein	
42	31184	33466	2283	+	0.407	internal virion protein	structural genes
43	33475	33957	483	+	0.387	endolysin	lysis
44	33972	39428	5457	+	0.405	internal virion protein	structural genes
45	39430	43908	4479	+	0.421	long tail fiber protein	structural genes
46	43919	44128	210	+	0.405	class II holin	lysis
47	44142	44438	297	+	0.374	hypothetical protein	
48	44435	44896	462	+	0.39	PhoB	auxiliary metabolism
49	44893	45285	393	+	0.399	hypothetical protein	
50	45285	45602	318	+	0.403	terminase small subunit	packaging genes
51	45602	47656	2055	+	0.412	terminase large subunit	packaging genes
52	47653	48066	414	+	0.43	hypothetical protein	
53	48066	48380	315	+	0.419	hypothetical protein	

Table S3. The genetic comparison of the phage vB_AmeP-R8W (R8W), vB_AspP-H4/4 (H4) and prokaryotic dsDNA virus (TS) (similarity >70%).

R8W genes No.	Start	End	Length	Putative function	Functional categories
17	7956	9203	1248	DnaB-like helicase	DNA replication and repair
19	9780	10451	672	thymidylate synthase	auxiliary metabolism
21	10920	11930	1011	ribonucleotide reductase, small chain	nucleotides metabolism
22	11906	13597	1692	ribonucleotide reductase, barrel domain	nucleotides metabolism
24	14009	14449	441	NTP-Ppase	auxiliary metabolism
25	14954	17266	2313	DNA polymerase	DNA replication and repair
27	17394	18275	882	hypothetical protein	
32	20532	20918	393	recombination endonuclease VII	DNA replication and repair
33	20915	23305	2391	DNA-dependent RNA polymerase	transcription
34	23332	23802	471	short tail fiber protein	structural genes
35	23778	24026	249	hypothetical protein	
36	24035	25573	1539	head to tail connector	structural genes
38	26473	27576	1104	putative major capsid protein	structural genes
43	33475	33957	483	endolysin	lysis
51	45602	47656	2055	terminase large subunit	packaging genes
52	47653	48066	414	hypothetical protein	
53	48066	48380	315	hypothetical protein	

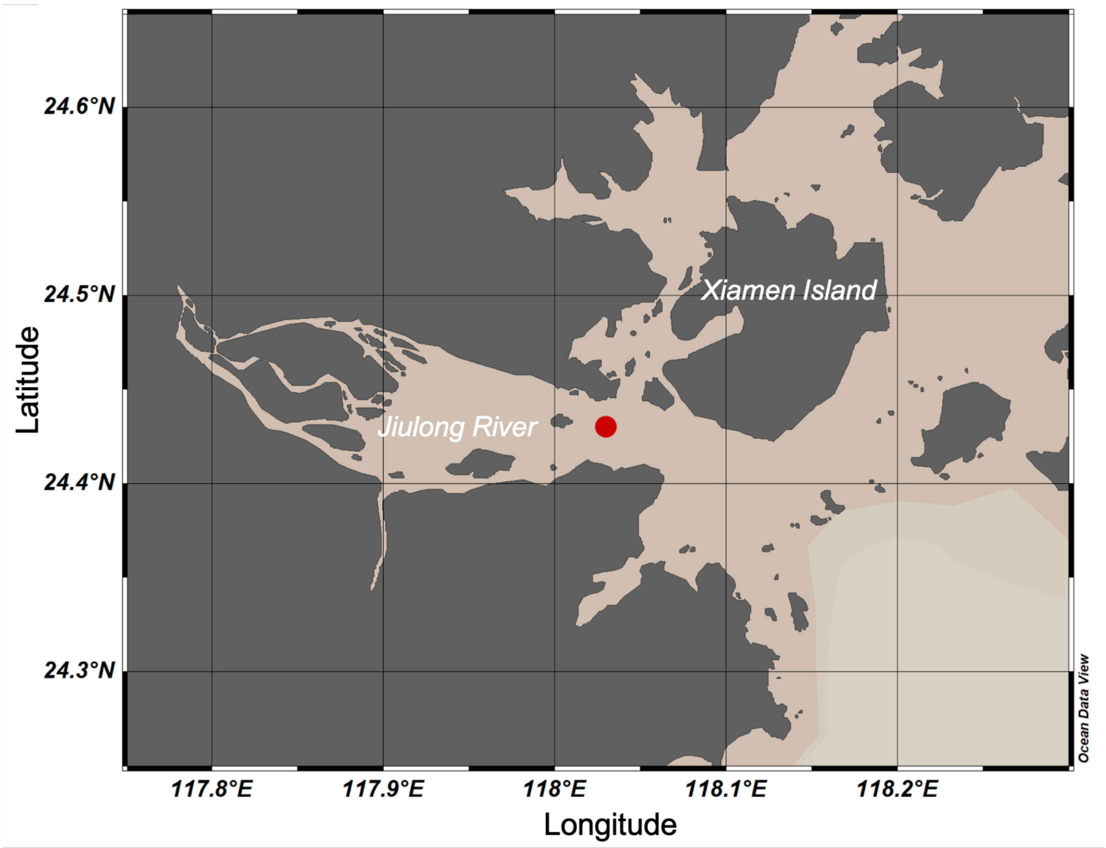

Figure S1. Station map indicating location where vB_AmeP-R8W (R8W) was isolated.

References

1. Baumann, L.; Baumann, P.; Mandel, M.; Allen, R.D. Taxonomy of aerobic marine Eubacteria. *J Bacteriol* **1972**, *110*, 402–429, doi:10.1128/jb.110.1.402-429.1972.

2. Yoon, J.; Kim, I.; Kang, K.H.; Oh, T.; Park, Y. *Alteromonas* marina sp. nov., isolated from sea water of the East Sea in Korea. *Int J Syst Evol Micr* **2003**, *53*, 1625–1630, doi:10.1099/ijs.0.02536-0.

3. Trappen, S.V.; Tan, T.; Yang, J.; Mergaert, J.; Swings, J. *Alteromonas stellipolaris* sp. nov., a novel, budding, prosthecate bacterium from Antarctic Seas, and emended description of the genus *Alteromonas*. *Int J Syst Evol Micr* **2004**, *54*, 1157–1163, doi:10.1099/ijs.0.02862-0.

4. Pujalte, M.; Sitjà-Bobadilla, A.; Álvarez-Pellitero, P.; Garay, E. Carriage of potentially fish-pathogenic bacteria in sparus aurata cultured in Mediterranean fish farms. *Dis Aquat Organ* **2003**, *54*, 119–126, doi:10.3354/dao054119.

5. Chiu, H.; Shieh, W.Y.; Lin, S.Y.; Tseng, C.; Chiang, P.; Wagner-Döbler, I. *Alteromonas tagae* sp. nov. and *Alteromonas simiduii* sp. nov., mercury-resistant bacteria isolated from a taiwanese estuary. *Int J Syst Evol Micr* **2007**, *57*, 1209–1216, doi:10.1099/ijs.0.64762-0.

6. Lopez-Lopez, A.; Bartual, S.G.; Stal, L.; Onyshchenko, O.; Rodriguez-Valera, F. Genetic analysis of housekeeping genes reveals a deep-sea ecotype of *Alteromonas macleodii* in the Mediterranean Sea. *Environ Microbiol* **2005**, *7*, 649–659, doi:10.1111/j.1462-2920.2005.00733.x.

7. Park, S.; Kang, C.-H.; Won, S.; Park, J.; Kim, B.; Yoon, J. *Alteromonas confluentis* sp. nov., isolated from the junction between the ocean and a freshwater spring. *Int J Syst Evol Micr* **2015**, *65*, 3603–3608, doi:10.1099/ijsem.0.000457.

8. Tvars-Martínez, E.; D'auria, G.; Rodríguez-Valera, F.; Sánchez-Porro, C.; Ventosa, A.; Joint, I.; Mühling, M. Biogeography of the ubiquitous marine bacterium *Alteromonas macleodii* determined by multilocus sequence analysis. *Mol Ecol* **2008**, *17*, 4092–4106, doi:10.1111/j.1365-294x.2008.03883.x.

9. Ivanova, E.P.; Ng, H.J.; Webb, H.K.; Kurilenko, V.V.; Zhukova, N.V.; Mikhailov, V.V.; Ponamoreva, O.N.; Crawford, R.J. *Alteromonas australica* sp. nov., isolated from the Tasman Sea. *Anton Leeuw Int J G* **2013**, *103*, 877–884, doi:10.1007/s10482-012-9869-x.

10. Ivanova, E.P.; Bowman, J.P.; Lysenko, A.M.; Zhukova, N.V.; Gorshkova, N.M.; Sergeev, A.F.; Mikhailov, V.V. *Alteromonas addita* sp. nov. *Int J Syst Evol Micr* **2005**, *55*, 1065–1068, doi:10.1099/ijs.0.63521-0.

11. Shi, X.; Wu, Y.; Jin, X.; Wang, C.; Xu, X. *Alteromonas lipolytica* sp. nov., a poly-beta-hydroxybutyrate-producing bacterium isolated from surface seawater. *Int J Syst Evol Micr* **2017**, *67*, 237–242, doi:10.1099/ijsem.0.001604.

12. Sun, C.; Xamxidin, M.; Wu, Y.; Cheng, H.; Wang, C.-S.; Xu, X. *Alteromonas alba* sp. nov., a marine bacterium isolated from seawater of the west Pacific Ocean. *Int J Syst Evol Micr* **2018**, *69*, 278–284, doi:10.1099/ijsem.0.003151.

13. Ivanova, E.P.; López-Pérez, M.; Zabalos, M.; Nguyen, S.H.; Webb, H.K.; Ryan, J.; Lagutin, K.; Vyssotski, M.; Crawford, R.J.; Rodriguez-Valera, F. Ecophysiological diversity of a novel member of the genus *Alteromonas*, and description of *Alteromonas mediterranea* sp. nov. *Anton Leeuw Int J G* **2015**, *107*, 119–132, doi:10.1007/s10482-014-0309-y.

14. Sass, A.M.; Sass, H.; Coolen, M.J.L.; Cypionka, H.; Overmann, J. Microbial communities in the chemocline of a hypersaline deep-sea basin (Urania Basin, Mediterranean Sea). *Appl Environ Microb* **2001**, *67*, 5392–5402, doi:10.1128/aem.67.12.5392-5402.2001.

15. Matsuyama, H.; Minami, H.; Sakaki, T.; Kasahara, H.; Baba, S.; Ishimaru, S.; Hirota, K.; Yumoto, I. *Alteromonas gracilis* sp. nov., a marine polysaccharide-producing bacterium. *Int J Syst Evol Micr* **2015**, *65*, 1498–1503, doi:10.1099/ijs.0.000127.

16. Jin, H.M.; Kim, K.H.; Jeon, C.O. *Alteromonas naphthalenivorans* sp. nov., a polycyclic aromatic hydrocarbon-degrading bacterium isolated from tidal-flat sediment. *Int J Syst Evol Micr* **2015**, *65*, 4208–4214, doi:10.1099/ijsem.0.000563.

17. Sinha, R.K.; Krishnan, K.P.; Singh, A.; Thomas, F.A.; Jain, A.; Kurian, P.J. *Alteromonas pelagimontana* sp. nov., a marine exopolysaccharide-producing bacterium isolated from the southwest Indian ridge. *Int J Syst Evol Micr* **2017**, *67*, 4032–4038, doi:10.1099/ijsem.0.002245.

18. Yoon, J.; Yeo, S.; Oh, T.; Park, Y. *Alteromonas litorea* sp. nov., a slightly halophilic bacterium isolated from an intertidal sediment of the Yellow Sea in Korea. *Int J Syst Evol Micr* **2004**, *54*, 1197–1201, doi:10.1099/ijs.0.63079-0.

19. Park, S.; Choi, S.J.; Park, J.; Yoon, J. *Alteromonas aestuariivivens* sp. nov., isolated from a tidal flat. *Int J Syst Evol Micr* **2017**, *67*, 2791–2797, doi:10.1099/ijsem.0.002023.

20. Morris, J.J.; Kirkegaard, R.; Szul, M.J.; Johnson, Z.I.; Zinser, E.R. Facilitation of robust growth of *Prochlorococcus* colonies and dilute liquid cultures by "helper" heterotrophic bacteria. *Appl Environ Microb* **2008**, *74*, 4530–4534, doi:10.1128/aem.02479-07.

21. Martínez-Checa, F.; Béjar, V.; Llamas, I.; Moral, A. del; Quesada, E. *Alteromonas hispanica* sp. nov., a polyunsaturated-fatty-acid-producing, halophilic bacterium isolated from Fuente De Piedra, southern Spain. *Int J Syst Evol Micr* **2005**, *55*, 2385–2390, doi:10.1099/ijs.0.63809-0.

22. Vandecandelaere, I.; Nercessian, O.; Segaert, E.; Achouak, W.; Mollica, A.; Faimali, M.; Vos, P.D.; Vandamme, P. *alteromonas genovensis* sp. nov., isolated from a marine electroactive biofilm and emended description of *Alteromonas macleodii* Baumann et al. 1972 (Approved Lists 1980). *Int J Syst Evol Micr* **2008**, *58*, 2589–2596, doi:10.1099/ijs.0.65691-0.

Supplementary Data 1. Results of R8W PhageTerm Analysis

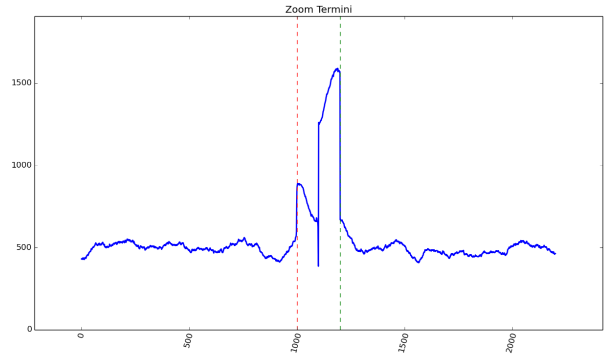

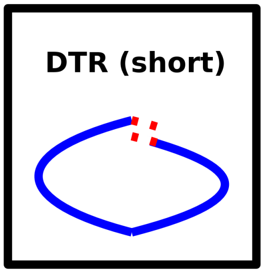

PhageTerm Method

Ends	Left (red)	Right (green)	Permuted	Orientation	Class	Type
Redundant	31181	31382	No	NA	DTR (short)	T7

Direct Terminal Repeats: 202 bp

Strand	Location	T	pvalue
+	31181	0.48	6.63e-55
	27210	0.05	1.00e+00
	31495	0.05	1.00e+00
	27057	0.05	1.00e+00
	12345	0.05	8.19e-01
-	31382	0.62	9.00e-141
	12063	0.06	1.00e+00
	17302	0.05	1.00e+00
	31956	0.05	1.00e+00
	3190	0.05	1.00e+00

T (Start. Pos. Cov. / Whole Cov.)

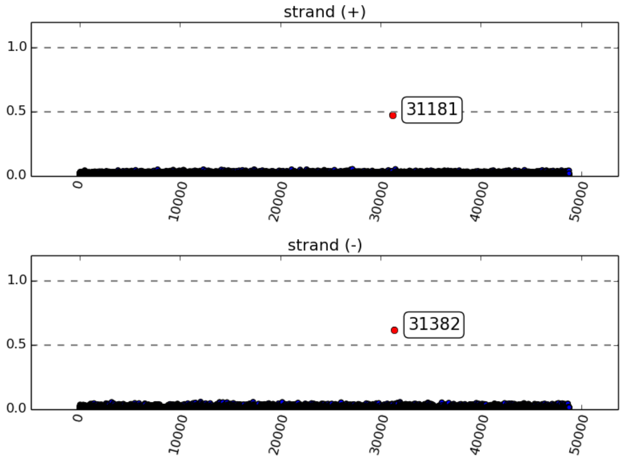

Li's Method

Packaging	Termini	Forward	Reverse	Orientation
COS	Fixed	Obvious Termini	Obvious Termini	Reverse

Direct Terminal Repeats: 202 bp

Strand	Location	SPC	R
+	31181	306	18.0
	12345	17	-
	21138	16	-
	43422	15	-
	17408	15	-
-	31382	924	66.0
	28012	14	-
	31956	13	-
	31410	13	-
	12063	13	-

SPC

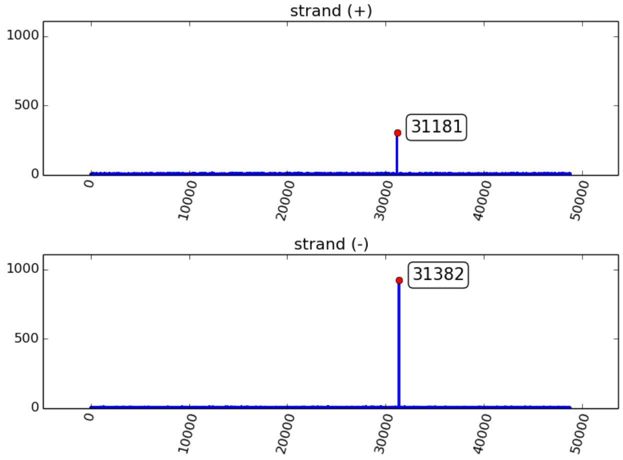

Analysis Methodology

PhageTerm software uses raw reads of a phage sequenced with a sequencing technology using random fragmentation and its genomic reference sequence to determine the termini position. The process starts with the alignment of NGS reads to the phage genome in order to calculate the starting position coverage (SPC), where a hit is given only to the position of the first base in a successfully aligned read (the alignment algorithm uses the lenght of the seed (default: 20) for mapping and does not accept gap or missmatch to speed up the process). Then the program apply 2 distinct scoring methods: i) a statistical approach based on the Gamma law; and ii) a method derived from LI and al. 2014 paper.

General set-up and mapping informations

**Phage Genome**	48825 bp
**Sequencing Reads**	1023457
**Mapping Reads**	95 %
OPTIONS	
Mapping Seed	*20*
Surrounding	*20*
Host Analysis	*No*

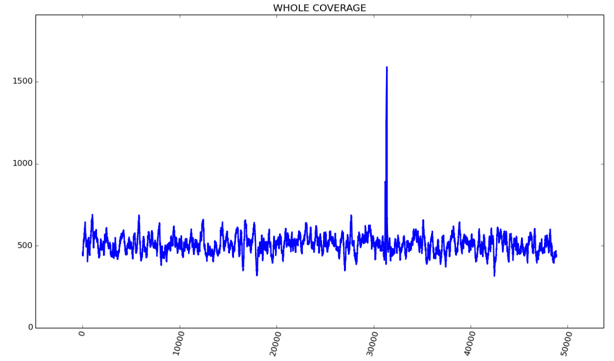

Highest peak of each side coverage graphics

Whole Coverage Zoom (Left) **Whole Coverage Zoom (Right)**

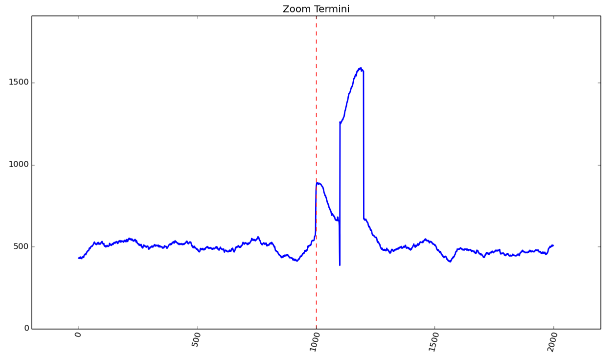

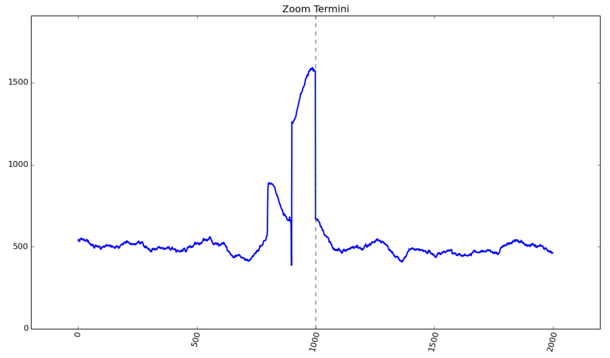

General controls information

**Whole genome coverage**	252	OK
**Weak genome coverage**	0.0 %	OK
**Reads lost during alignment**	4.9 %	OK

i) PhageTerm method

Reads are mapped on the reference to determine the starting position coverage (SPC) as well as the coverage (COV) in each orientation. These values are then used to compute the variable T = SPC / COV. The average value of T at positions along the genome that are not termini is expected to be 1/F, where F is the average fragment size. For the termini that depends of the packaging mode. Cos Phages: no reads should start before the terminus and therefore X=1. DTR phages: for N phages present in the sample, there should be N fragments that start at the terminus and N fragments that cover the edge of the repeat on the other side of the genome as a results T is expected to be 0.5. Pac phages: for N phages in the sample, there should be N/C fragments starting at the pac site, where C is the number of phage genome copies per concatemer. In the same sample N fragments should cover the pac site position, T is expected to be (N/C)/(N+N/C) = 1/(1+C). To assess whether the number of reads starting at a given position along the genome can be considered a significant outlier, PhageTerm first segments the genome according to coverage using a regression tree. A gamma distribution is then fitted to SPC for each segment and an adjusted p-value is computed for each position. Finally if several significant peaks are detected within a small sequence window (default: 20bp), their T values are merged.

**Nearby Termini (Forward / Reverse)**	2 / 0	Peaks localized 20 bases around the maximum

ii) Li's method

The second approach is based on the calculation and interpretation of three specific ratios R1, R2 and R3 as suggested in a previous publication from Li et al. 2014. The first ratio, is calculated as follow: the highest starting frequency found on either the forward or reverse strands is divided by the average starting frequency, R1 = (highest frequency/average frequency). Li's et al. have proposed three possible interpretation of the R1 ratio. First, if R1 < 30, the phage genome does not have any termini, and is either circular or completely permuted and terminally redundant. The second interpretation for R1 is when 30 ≤ R1 ≥ 100, suggesting the presence of preferred termini with terminal redundancy and apparition of partially circular permutations. At last if R1 > 100 that is an indication that at least one fixed termini is present with terminase recognizing a specific site. The two other ratios are R2 and R3 and the calculation is done in a similar manner. R2 is calculated using the highest two frequencies (T1-F and T2-F) found on the forward strand and R3 is calculated using the highest two frequencies (T1-R and T2-R) found on the reverse strand. To calculate these two ratios, we divide the highest frequency by the second highest frequency T2. So R2 = (T1-F / T2-F) and R3 = (T1-R / T2-R). These two ratios are used to analyze termini characteristics on each strand taken individually. Li et al. suggested two possible interpretations for R2 and R3 ratios combine to R1. When R1 < 30 and R2 < 3, we either have no obvious termini on the forward strand, or we have multiple preferred termini on the forward strand, if 30 ≤ R1 ≤ 100. If R2 > 3, it is suggested that there is an obvious unique termini on the forward strand. The same reasoning is applicable for the result of R3. Combining the results for ratios found with this approach, it is possible to make the first prediction for the viral packaging mode of the analyzed phage. A unique obvious termini present at both ends (both R2 and R3 > 3) reveals the presence of a COS mode of packaging. The headful mode of packaging PAC is concluded when we have a single obvious termini only on one strand. A whole coverage around 500X is needed for this method to be reliable.

**Nearby Termini (Forward / Reverse)**	3 / 1	Peaks localized 20 bases around the maximum
**R1 - highest freq./average freq.**	361	At least one fixed termini is present with terminase recognizing a specific site.
**R2 Forw - highest freq./second freq.**	18	Unique termini on the forward strand.
**R3 Rev - highest freq./second freq.**	66	Unique termini on the reverse strand.
